# Knowledge and Awareness of Type 2 Diabetes Complications in Conflict-Affected Regions: A Cross-Sectional Study in Homs, Syria

**DOI:** 10.7759/cureus.68686

**Published:** 2024-09-05

**Authors:** Noor Albawab, Batol Junbolat, Aya Almohamad, Kefah Albawab, Sedra Mando, Rama Asaad, Shahd Alhayek, Adel Habib, Mohammed Ahmed Albakoush, Mosa Shibani, Yaser Abas, Abdullah Alhouri

**Affiliations:** 1 Endocrinology and Metabolism, AL-Baath University Hospital, Homs, SYR; 2 Internal Medicine, Al-Baath University, Homs, SYR; 3 Internal Medicine, Al-Baath University Hospital, Homs, SYR; 4 General Medicine, Syrian Private University, Damascus, SYR; 5 Internal Medicine, Aneurin Bevan University Health Board, Wales, GBR; 6 Emergency Medicine, Royal Berkshire Hospital, Reading, GBR; 7 Medicine, Syrian Private University, Damascus, SYR; 8 Endocrinology and Metabolism, Al-Baath University Hospital, Homs, SYR

**Keywords:** awareness, diabetes mellitus, homs, knowledge, syria

## Abstract

Background

Diabetes mellitus is a significant global health problem characterized by high blood sugar levels due to either a lack of insulin or insensitivity to it. Concerns about diabetes complications are growing both globally and locally, making effective preventive measures crucial to tackle these complications. This research aimed to determine the level of knowledge and awareness regarding type 2 diabetes complications among patients in Homs, Syria, during the ongoing conflict.

Methods

A cross-sectional study was conducted among 1016 diabetic patients aged 16 years and above attending internal medicine and diabetes clinics in Homs City through a structured questionnaire administered via social media, telephone interviews, and paper forms. The data collected were analyzed using SPSS version 23.0 (IBM Corp., Armonk, USA). Descriptive statistics were used to summarize the demographic characteristics. In contrast, inferential statistics such as the chi-square test were used to test for associations between different variables with p-value < 0.05 as the significance level.

Results

The study found that 478 (47.1%) of participants were male, and 538 (53%) were female. The majority 652 (64.2%) were between 46 and 70 years old, and 422 (41.5%) had primary-level education. Most were married 750 (73.8%) and lived in urban areas 856 (84.2%). Diabetes knowledge varied significantly by age, education, occupation, and residence. Housewives and those with lower education levels had the least knowledge, while urban residents and those with 1-5 years since diagnosis had better awareness. Individuals with relatives in the medical field had higher knowledge levels. Results indicate that gender did not significantly influence diabetes knowledge (p = 0.19), while younger adults showed poorer awareness compared to older age groups (p < 0.05). Higher education levels were associated with better knowledge (p < 0.05), and a family history of diabetes correlated with greater awareness of complications. Occupation significantly impacted knowledge, with employed individuals and urban residents demonstrating higher awareness levels (p < 0.05). Diabetes knowledge peaked during the 6-10 year disease duration (p < 0.05).

Conclusion

This study assesses diabetes knowledge and management practices among 1016 participants in Homs, Syria, focusing on demographic and socioeconomic factors. Our findings underscore the need for targeted educational initiatives to improve diabetes management, particularly among younger, less educated, and rural populations in Homs. The analysis reveals significant knowledge gaps about diabetes across different demographics in Homs, highlighting the need for targeted educational programs, especially for younger individuals, the less educated, and rural residents. The study emphasizes the importance of education and medical practice in understanding diabetes, particularly in conflict-affected areas

## Introduction

Diabetes mellitus, a metabolic disorder, is characterized by elevated blood glucose levels due to the disablement of insulin secretion, defective insulin action, or both [1]. The pathophysiology of diabetes, which involves multiple hormones such as insulin, glucagon, and growth hormone, is complex and crucial to understand for effective prevention and management (2,3). The disorder is classified into several types, with type 1 and type 2 being the most significant [2,3].

In 2021, the global adult diabetes cases reached a staggering 537 million, with projections soaring to 643 million by 2030 and a daunting 783 million by 2045. Diabetes claimed 6.7 million lives in 2021 alone [4]. The Middle East and North Africa bore a significant burden, with 73 million adults grappling with diabetes in 2021, a number expected to surge to 95 million by 2030 and a staggering 136 million by 2045. Syria, in particular, had 1,484.2 thousand adult diabetes cases in 2021, a figure projected to escalate to approximately 2,596.0 thousand by 2030 and a staggering 3,760.1 thousand by 2045 [4]. These numbers underscore the escalating global and regional impact of diabetes, underscoring the urgent need for effective prevention and management.

The autoimmune system attacks the pancreatic islet's insulin-producing beta cells, leading to an acute reduction in insulin secretion, known as Type 1 diabetes [5]. This kind of diabetes accounts for about 5-10% of cases [5]. The primary cause is when the body starts destroying itself by creating autoantibodies against specific beta-cell antigens located thereon, thus participating in their destruction [5]. However, type 2 diabetes differs from type 1 because patients make insulin but fail to respond adequately due to β-cell resistance or deficiency alongside glucotoxicity following sustained hyperglycemia [6].

High blood sugar levels in diabetes can cause short-term and long-term problems. Long-term issues involve damage to big blood vessels (macrovascular) and small blood vessels (microvascular). Short-term problems include diabetic ketoacidosis (DKA) and hyperosmolar hyperglycemic syndrome (HHS) [7-9]. DKA happens when there is not enough insulin and requires hospital care because it can be life-threatening [8]. HHS occurs when blood sugar is very high with little ketosis. This is a considerable risk for people with type 2 diabetes and requires hospital treatment to fix body system problems [9].

Macrovascular issues like hardening of the arteries can lead to heart disease, a significant cause of morbidity and mortality in diabetes [10]. However, it's crucial to remember that these problems are largely preventable with careful diabetes management. People with diabetes also have about twice the risk of stroke compared to those without diabetes [11]. These problems show how diabetes impacts many body systems. This highlights the need to manage diabetes carefully and take steps to prevent complications, offering hope and motivation for effective diabetes management.

Diabetes-induced peripheral arterial disease is responsible for causing critical limb ischemia, insulin resistance, and hyperglycemia, which bring about metabolic changes that are damaging to muscles, thereby retarding their healing [12]. Among people with diabetes, the risk of developing kidney failure is ten times greater than in those without the condition; also, individuals have a 3 in 10 chance of losing some level of sight because of this disease [13]. Diabetic neuropathy includes peripheral neuropathy, autonomic neuropathy, focal neuropathy, and proximal neuropathy, further broadening its complications spectrum [14].

During emergencies like wars or natural disasters, individuals with lifelong illnesses such as diabetes often experience psychological trauma that may lead to neglecting their health. The lack of accessible information on diabetes complications and management in conflict situations further exacerbates the burden on both patients and healthcare providers, especially in developing countries where national disaster response plans are often lacking and healthcare resources are already strained [15]. This research aimed to determine the level of knowledge and awareness regarding type 2 diabetes complications among patients in Homs, Syria, during the ongoing conflict. To our knowledge, no published data currently exists on this topic for patients in Homs.

## Materials and methods

Study design and setting

This is a cross-sectional study conducted in the internal medicine clinic (private or public or both, urban and rural areas) and diabetes clinic at Al Arman Center in Homs to assess awareness and knowledge about diabetes among diabetic patients in the Syrian community in Homs. Diabetic type 1 and 2 patients aged 16 years and above were eligible to be included. Data was collected between 23rd June and 1st October 2023.

Ethical considerations

The study was approved by the institutional review board (IRB) of Al-Baath University Hospital. The relevant guidelines and regulations performed all procedures. The questionnaire elucidated the objectives of the study in detail. Before completing it, participants gave their informed consent voluntarily. Anonymity and confidentiality of information were also ensured through coding, where only investigators knew each subject's identity.

Statistical analysis

Descriptive statistics using Statistical Package for Social Science (SPSS) version 23.0 (IBM Corp., Armonk, USA) were employed to analyze demographic characteristics frequencies among respondents and their responses distribution pattern towards different question items on the questionnaire. Inferential tests such as chi-square were conducted to establish associations between knowledge and awareness about diabetes and other study variables. The significance level is p-value <0.05.

Data collection and procedure

This study utilized a convenience sample comprising 1016 participants, selected through a structured and validated questionnaire (see Appendices) [16]. Data collection employed diverse methods, including face-to-face interviews, telephone surveys, and other modalities. Specifically, participants were reached through social networking sites such as Facebook, Instagram, and WhatsApp, as well as paper-based questionnaires distributed among patients in private clinics. Additionally, telephone interviews were conducted with individuals registered at the diabetes clinic at Al Arman Center, with the investigator providing assistance to illiterate patients by explaining questions and completing the questionnaire on their behalf. The questionnaire, which includes 28 items on diabetes complications, assesses participants' awareness by summing their correct answers. With a mean score of 15 correct answers and a range between 5 and 25, participants were classified based on their performance: those who correctly answered 15 or more questions were categorized as having awareness. In contrast, those who answered fewer than 15 questions were categorized as lacking awareness.

## Results

The research sample included 1016 participants. Of these, 478 (47.1%) were male, while 538 (53%) were female. Age-wise, 46-70 years was the highest age group with a percentage of 652 (64.2%); 31-45 years had 145 (14.3%), over 70 years took 128 (12.6%), whereas 18-30 years recorded 9%. With respect to the level of education attained by individuals who took part in the survey, those who had gone up to primary school were 422 (41.5%), bachelor's degree holders were 255 (25.1%), and secondary school leavers accounted for 188 (18.5%). In contrast, illiterate people constituted 133 (13.1%), and post-graduate degree owners constituted 18 (1.8%). The marital status distribution revealed that most people were married at 750 (73.8%), widowed came next at 138 (13.6%), and single individuals occupied the third place at 116 (11.4%). At the same time, the divorced had the lowest percentage, which stood at 12 (1.2%) (Table 1).

**Table 1:** Participant demographics and socioeconomic properties

Variable (N =1016)	Frequency	Percent
Gender		
Male	478	47.1%
Female	538	53%
Age; year		
18–30	91	9%
31–45	145	14.3%
46–70	652	64.2%
>70	128	12.6%
Level of education		
Illiterate	133	13.1%
Primary school	422	41.5%
secondary school	188	18.5%
Bachelor's degree	255	25.1%
Post graduate	18	1. 8%
Marital status		
Married	750	73.8%
Divorced	12	1.2%
Widowed	138	13.6%
Single	116	11.4%
Economic status		
Low	278	27.7%
Moderate	523	51.9%
Good	178	17.5%
Very good	37	3.6%
Work status		
Don't work	306	30.1%
Part-time work	138	13.6%
Full-time work	180	17.7%
Student (not worker)	41	4%
Housekeeper	351	34.5%
Residence		
Rural	160	15.8%
Urban	856	84.2%
Duration of Diabetes; year		
1-5	294	28.9%
6-10	201	19.8%
> 10	521	51.3%
Smoking		
Yes	310	30.5%
No	706	69.5%

Regarding their economic well-being, most respondents considered themselves moderate, with 523 (51.9%). However, only 37 (3.6%) were having very good economic status. In contrast, others said they had good economic standards 178 (17.5%), and quite a number also admitted having low-income status 278 (27.7%). Work status showed a considerable proportion of people who are not employed; this category recorded the highest figures, followed by housewives who took up 34.5%, then came full-time workers constituting 180 (17.7%), while part-time working individuals formed 138 (13.6%). Lastly, students comprised only 41 (4%). Most participants lived in urban areas, accounting for 856 (84.2%) (Table 1).

Regarding the duration of suffering from diabetes among those interviewed for this study, the majority had been diabetic patients for more than ten years, representing 521 (51.3%), 1-5 years 294 (28.9%), followed by 6-10 years at 201 (19.8%). Concerning smoking habits, it was found that 310 (30.5%) were smokers. At the same time, 706 (69.5) took up the nonsmokers group (Table 1).

The research also sought to determine if the respondents were knowledgeable about managing diabetes and its complications (Table 2). Most people stated the normal fasting blood sugar is between 70-110mg/dl, but only (15) 1.5% of the responses were less than 70mg/dl; there were quite a number who did not know the normal value 146 (14.8%). Regarding symptom awareness, our data revealed that thirst ranked highest at 637 (62.7%), followed by frequent urination at 694 (68.3%), blurring of vision at 402 (39.6%), general body weakness at 591 (58.2%), and dry mouth at 604 (59%). At the same time, confusion had the lowest score 67 (6.6%).

**Table 2:** Awareness of DM complications among type 2 DM patients in Homs, Syria DM: diabetes mellitus

Variables	Number	Percent
What is the normal fasting blood sugar level?		
<70 mg/dl	15	1.5%
70—110 mg/dl	367	36.1%
> 126 mg/dl	488	48.%
Don't know	146	14.8%
What are the most common symptoms of high blood sugar?		
Increased thirst	637	62.7%
Frequent urination	694	68.3%
Blurring of vision	402	39.6%
Weakness	591	58.2%
Dry mouth	604	59%
Confusion	67	6.6%
What are the most common symptoms of low blood sugar?		
Palpitation	386	38%
Tremor	578	56.9%
Sweating	484	47.6%
Blurring of vision	321	31.6%
Decreased coordination	390	38.4%
Don't know	196	19.3%
Which of the following complications can happen when diabetes is not well controlled?		
Diabetic foot	525	51.7%
Eye complications	641	63.1%
Heart complications	359	35.3%
Neuropathy	580	57.1%
Renal complications	415	40.8%
Stroke	151	14.9%
Teeth decay	166	16.3%
Hypertension	296	29.1%
Sexual dysfunction	237	23.3%
Don't know	146	14.4%
Can dietary modification prevent diabetic complication?		
Yes	899	88.5%
No	56	5.5%
Don't know	61	6%
Can smoking/and alcohol stopping prevent diabetic complication?		
Yes	606	59.6%
No	135	13.3%
Don't know	275	27.1%
Is physical work or exercise help to prevent diabetes complication?		
Yes	775	76.3%
No	88	8.7%
Don't know	153	15.1%
If you are beginning to have a low blood sugar you should?		
Exercise	22	2.2%
Lie down and rest	205	20.2%
Drink some juice	757	74.5%
Take rapid-acting insulin	52	5.1%
Don't know	153	15.1%
What you should do when your blood sugar is raised?		
Dietary modification	360	35.4%
Physical exercise	197	19.4%
Lowering stress	138	13.7%
Take insulin	299	29.4%
Take his/her normal drug	496	48.8%
Go to doctor	220	21.6%
Don't know	92	9.1%

On complications of uncontrolled diabetes (Table 2), 525 ( 51.7% ) mentioned diabetic foot, 641 (63.1%) eye complications, 359 (35.3%) heart complications, 580 (57.1%) neuropathy, 415 (40.8%) renal complications, 151 (14.9%) stroke, 166 (16.3%) teeth decay, 296 (29.1% )hypertension, 237 (23.3%) sexual dysfunction, and 146 (14.4%) didn't know. Most participants 899 (88.5%) believed dietary modification prevents complications, while 56 (5.5%) disagreed, and 61 (6%) didn't know. Similarly, 606 (59.6%) thought stopping smoking and alcohol helps, 135 (13.3%) disagreed, and 275 (27.1%) were unsure. Regarding physical exercise, 775(76.3%) agreed it prevents complications, 88 (8.7%) disagreed, and 153 (15.1%) didn't know. For low blood sugar, 22 (2.2%) suggested exercise, 205 (20.2%) lying down, 757 (74.5%) drinking juice, 52 (5.1%) taking insulin, and 153 (15.1%) didn't know. For raised blood sugar, 360 (35.4%) recommended dietary modification, 197 (19.4%) exercise, 138 (13.7%) lowering stress, 299 (29.4%) insulin, 496 (48.8%) regular medication, 220 (21.6%) seeing a doctor, and 92 (9.1%) not knowing.

The study examined the association between various demographic factors and individuals' knowledge status regarding diabetes (Table 3). Among the 1016 participants, significant associations were observed between knowledge status and several demographic variables. Regarding sex, there was no statistically significant difference in knowledge status between males and females (p = 0.19). However, age showed a significant association, with younger age groups (18-30 years and 31-45 years) demonstrating poorer knowledge compared to older age groups (46-70 years and > 70 years) (p <0.05). Education also played a significant role, with higher education levels correlating with better knowledge about diabetes. Illiterate individuals showed the poorest knowledge status, while those with Master's or Doctorate degrees exhibited the highest level of knowledge (p <0.05). Occupation was another influential factor, with students and housewives demonstrating lower understanding than those with full-time or part-time jobs (p < 0.05). Similarly, rural residents tended to have poorer knowledge than urban residents (p < 0.05).

**Table 3:** Factors associated with awareness of DM complications among DM patients in Homs, Syria DM: diabetes mellitus

Variable (N =1016)	Knowledge status		P-value
	Poor N (%)	Good N (%)	
Sex			0.19
Male	274 (57.3%)	204 (42.7%)	
Female	331 (61.5%)	207 (38.5%)	
Age; year			<0.05
18–30	37 (40.7%)	54 (59.3%)	
31–45	89 (61.4%)	56 (38.6%)	
46–70	398 (61 %)	254 (39%)	
>70	81 (63.3%)	47(36.7%)	
Level of Education			<0.05
Illiterate	95 (71.4%)	38 (28.6%)	
Primary School	268 (63.5%)	154 (36.5%)	
Secondary School	109 (58%)	79 (42%)	
Bachelor's Degree	126 (49.4%)	129 (50.6%)	
Master's Degree or Doctorate Degree	7 (39%)	11 (61.1%)	
Occupation			<0.05
Not Work	191 (62.4%)	115 (37.6%)	
Full-Time Job	67 (48.6%)	71 (51.4%)	
Part-Time Job	102 (56.7%)	78 (43.3%)	
Student	17 (41.5%)	24 (58.5%)	
Housewife	228 (65%)	123 (35%)	
Residence			<0.05
Rural	99 (62.3)	60 (37.7)	
Urban	108 (44.4)	135 (55.6)	
Duration of Diabetes; years			<0.05
1-5	197 (67%)	97 (33%)	
6-10	112 (55.7%)	89 (44.2%)	
> 10	296 (56.8%)	225 (43.1%)	
Family History of Diabetes			0.12
Yes	438 (58.1%)	316 (41.9%)	
No	167 (63.7%)	95 (36.3%)	
Income			0.21
low	173 (62.2%)	105 (37.8%)	
Middle	317 (60.6%)	206 (39.4%)	
Good	94 (52.8%)	84 (47.2%)	
Excellent	21 (56.8%)	16 (43.2%)	
Do You Belong to The Medical Field			<0.05
Yes	25 (36.76%)	43 (63.24%)	
No	580 (61.18%)	368 (38.82%)	
Does Anyone from Your Family Belong to The Medical Field			<0.05
Yes	247 (55.1%)	201 (44.9%)	
No	358 (63%)	210 (37%)	

Diverse outcomes of living with diabetes are dependent on when the patient was diagnosed. Those diagnosed between one and five years ago possess more knowledge than those diagnosed earlier (p < 0.05). It is worth noting that people from families where some members are doctors have a better understanding of this condition (p < 0.05). Additionally, income and family medical history had no bearing on how much people knew about diabetes in this research (Table 3).

## Discussion

Diabetes mellitus, a chronic metabolic disorder characterized by hyperglycemia, poses a significant global health challenge. Effective management and control of diabetes largely depend on patients' knowledge and understanding of the disease, including its complications, self-care practices, and the impact of lifestyle choices. This study aimed to assess the knowledge and management practices of diabetic patients, exploring the influence of various demographic and socioeconomic factors on their understanding and behavior regarding diabetes care.

In this study, gender did not significantly influence diabetes knowledge (p = 0.19). This finding is consistent with similar studies where no significant gender differences were reported in diabetes awareness and management knowledge [17]. However, studies in Brazil and China found that women were more aware of diabetes than men [18, 19]. Contrarily, research from Syria, Ethiopia, Saudi Arabia, and the UAE observed a significant association between gender and diabetes awareness, with males having more awareness than females [16, 20-22]. This disparity can be attributed to cultural influences, where men, spending more time outdoors, have more opportunities to learn new medical facts and discuss health issues with peers. In contrast, women, spending more time at home, might have fewer such opportunities.

Our study showed that younger age groups (18-30 years and 31-45 years) exhibited poorer knowledge compared to older age groups (46-70 years and > 70 years) (p < 0.05). This trend is corroborated by other Syrian and Ethiopian research indicating that older adults often have better knowledge about diabetes, but this differs from findings in Lebanon and Saudi Arabia, where younger people were the most aware [16, 17]. Our results can be explained by the fact that older patients have experienced more complications of the disease than younger patients, who have lived with the disease for fewer years [16, 20, 21]. Younger individuals may benefit from targeted educational interventions to bridge this knowledge gap.

The positive correlation between higher education levels and better diabetes knowledge is a common finding across numerous studies [23, 24]. Individuals with primary education or lower exhibited significantly poorer understanding than those with bachelor's degrees or higher (p < 0.05). This underscores the critical role of education in enhancing health literacy and the effectiveness of diabetes management programs.

Another notable finding from this study is that contrary to previous research conducted in Iran and among African Americans [25, 26], our results did not reveal a significant relationship between having a family history of diabetes and awareness of diabetes complications. This indicates that, in our study population, family history might not be a determining factor for diabetes knowledge. These findings highlight the need for further research to explore other potential influences on diabetes awareness and adopting preventive health practices.

Occupation significantly influences diabetes knowledge, with unemployed individuals and housewives demonstrating lower knowledge levels than their employed counterparts (p < 0.05). Employed individuals often have better access to health information and resources through workplace programs and social interactions. Conversely, unemployed individuals and housewives may need help with economic and social constraints limiting their exposure to health education. This finding aligns with research showing that employment status positively affects diabetes knowledge [20, 25]. A study in Bangladesh also found higher diabetes awareness among employees compared to housewives [27]. In developing countries like Syria, women, particularly housewives, often have less access to health information, reflecting broader socioeconomic and cultural barriers.

Urban residents demonstrated significantly better knowledge of diabetes compared to their rural counterparts (55.6% vs. 37.7%, p < 0.05). This finding is consistent with various studies highlighting disparities in healthcare access and education between urban and rural areas [22, 28-29]. The higher knowledge levels among urban residents suggest the need for improved healthcare outreach and education programs in rural communities to address these disparities. Enhanced efforts are necessary to bridge the healthcare access and knowledge gap between urban and rural areas.

Our findings indicate that diabetes awareness peaks during the disease's 6-10 years duration, with knowledge levels remaining stable for durations beyond 10 years (p < 0.05). This trend suggests that patients tend to gain more knowledge about diabetes as they live with the condition longer. Similar results have been reported in Saudi Arabia and Gambia studies, which also found a positive correlation between the length of time a patient has diabetes and their awareness of the disease [30, 31].

Contrary to expectations, income did not significantly affect diabetes knowledge in this study. This differs from some research that suggests higher socioeconomic status is often associated with better health literacy and disease management [16]. The lack of significant association here could be due to other overriding factors, such as educational level and access to healthcare resources, which might play a more critical role in this particular population.

The study's limitations include potential sampling bias, reliance on self-reported data, and exclusion of patients without access to clinics or communication tools, particularly in conflict-affected rural areas. The cross-sectional design limits causal inferences, and the study's focus on Homs City may not represent broader populations. 

## Conclusions

This study underscores the multifaceted nature of diabetes knowledge and the influence of demographic factors. By aligning with findings from other research, it validates the need for comprehensive, inclusive educational strategies to improve diabetes management. Public health initiatives should prioritize accessible education, especially for vulnerable groups identified in this study, to foster a more informed and health-conscious population. Targeted educational programs for younger individuals, rural residents, and those with lower educational attainment are essential. Moreover, leveraging family networks, especially those with medical backgrounds, could enhance community health education efforts.

## Appendices

**Table 4:** Knowledge and Awareness of Type 2 Diabetes Complications Questionnaire

1- Gender:
Male
Female
2- Age; year:
18–30
31–45
46–70
>70
3- Level of education:
Illiterate
Primary school
secondary school
Bachelor's degree
Post graduate
4- Marital status:
Married
Divorced
Widowed
Single
5- Economic status:
Low
Moderate
Good
Very good
6- Work status:
Don't work
Part-time work
Full-time work
Student (not worker)
Housekeeper
7- Residence:
Rural
Urban
8- Duration of Diabetes; year:
1-5
6-10
> 10
9- Smoking:
Yes
No
10- What is the normal fasting blood sugar level?
<70mg/dl
70—110mg/dl
> 126mg/dl
Don't know
11- What are the most common symptoms of high blood sugar?
Increased thirst
Frequent urination
Blurring of vision
Weakness
Dry mouth
Confusion
12- What are the most common symptoms of low blood sugar?
Palpitation
Tremor
Sweating
Blurring of vision
Decreased coordination
Don't know
13- Which of the following complications can happen when diabetes is not well controlled?
Diabetic foot
Eye complications
Heart complications
Neuropathy
Renal complications
Stroke
Teeth decay
Hypertension
Sexual dysfunction
Don't know
14- Can dietary modification prevent diabetic complication?
Yes
No
Don't know
15- Can Stop smoking/and alcohol stopping prevent diabetic complication?
Yes
No
Don't know
16- Is physical work or exercise help to prevent diabetes complication?
Yes
No
Don't know
17- If you are beginning to have a low blood should?
1.Exercise
2. Lie down and rest
3. Drink some juice
4. Take rapid-acting insulin
5. Don't know
18- What you should do when your blood sugar is raised?
1. Dietary modification
2. Physical exercise
3. Lowering stress
4.Take insulin
5. Take his/her normal drug
6. Go to doctor
7. Don't know
